# Self-Monitoring Symptoms in Glaucoma: A Feasibility Study of a Web-Based Diary Tool

**DOI:** 10.1155/2017/8452840

**Published:** 2017-05-04

**Authors:** Leanne McDonald, Fiona C. Glen, Deanna J. Taylor, David P. Crabb

**Affiliations:** Division of Optometry and Visual Sciences, School of Health Sciences, City, University of London, London EC1V 0HB, UK

## Abstract

*Purpose*. Glaucoma patients annually spend only a few hours in an eye clinic but spend more than 5000 waking hours engaged in everything else. We propose that patients could self-monitor changes in visual symptoms providing valuable between clinic information; we test the hypothesis that this is feasible using a web-based diary tool. *Methods*. Ten glaucoma patients with a range of visual field loss took part in an eight-week pilot study. After completing a series of baseline tests, volunteers were prompted to monitor symptoms every three days and complete a diary about their vision during daily life using a bespoke web-based diary tool. Response to an end of a study questionnaire about the usefulness of the exercise was a main outcome measure. *Results*. Eight of the 10 patients rated the monitoring scheme to be “valuable” or “very valuable.” Completion rate to items was excellent (96%). Themes from a qualitative synthesis of the diary entries related to behavioural aspects of glaucoma. One patient concluded that a constant focus on monitoring symptoms led to negative feelings. *Conclusions*. A web-based diary tool for monitoring self-reported glaucoma symptoms is practically feasible. The tool must be carefully designed to ensure participants are benefitting, and it is not increasing anxiety.

## 1. Introduction

Whilst the clinical and biological mechanisms of glaucoma are well explored, the impact of glaucoma on an individual's well-being has been relatively understudied [1, 2]. Patient-reported outcome measures (PROMs) estimate perceived health status, functional status, or health-related quality of life. PROMs, often administered as questionnaires, have been used to assess the effect of glaucoma on the quality of life in research studies for some time [3, 4]. PROMs are starting to be used as end points in clinical trials of treatments for glaucoma [5]. Such use of PROMs is a positive step because they directly assess the impact of symptoms of disease on a patient, certainly as they perceive it themselves. To date, PROMs are not used in regular clinical management of patients with glaucoma. Yet the benefits for this idea have been speculated upon, and PROMs are being increasingly used in the clinical management of other conditions [6, 7].

In the United Kingdom (UK), there are more than one million hospital visits a year for glaucoma [8]; clinicians likely have inadequate time and resources to cope with these visits. Moreover, patients likely do not get the opportunity to discuss their psychological well-being or the functional impact of their glaucoma at these visits. This is a pity because better between clinic visit information and time for patient/clinician interaction may lead to better glaucoma management [9]. At the same time, patients spend only a few hours a year in the eye clinic having their glaucoma monitored but they spend more than 5000 waking hours each year engaged in everything else [10]. This statistic suggests that there should be time for patients to potentially self-monitor their symptoms in between clinic visits. Self-monitoring approaches have proved effective in other chronic conditions such as type 2 diabetes [11]; these methods might be useful for people with glaucoma, and this is the main idea explored in this study.

In this work, we explore how people with glaucoma might self-monitor changes in visual symptoms with the aim of making them more engaged in their “glaucoma journey.” We also examine how self-monitoring may be influenced by personality traits. We specifically test the hypothesis that a group of volunteer patients will be sufficiently motivated to regularly self-report on their symptoms; we examine the feasibility of this using a web-based diary tool.

## 2. Materials and Methods

Participants responded to an invitation to take part in the study from a patient-based charitable organisation (International Glaucoma Association—http://www.glaucoma-association.com). The study was a prospective mixed-method feasibility study which took place over eight weeks in 2015.

Ten participants were recruited from different glaucoma clinics across England; all had a clinical diagnosis of primary open-angle glaucoma (POAG) with at least a five-year treatment history. Participants were asked to respond if they had glaucoma alone and no other ocular disease other than prior uncomplicated cataract surgery.

The study was approved by a Research and Ethics Committee (City, University of London, School of Health Sciences) and adhered to the tenets of the Declaration of Helsinki. Data was anonymised and stored in a secure location. All participants gave their informed written consent prior to taking part.

### 2.1. Pretesting

Participants were asked to attend the university to complete a series of pretest measures to confirm their eligibility for the study. A Mini-Mental State Examination was used to exclude people with any measureable cognitive impairment. Participants then underwent an examination of their vision by a qualified optometrist (DJT). This examination included refraction, measurement of contrast sensitivity (CS), visual acuity (VA), and slit-lamp examination on both eyes. An examination of the visual field (VF) confirmed that all participants had measureable VF loss in at least one eye. VFs were measured (Swedish Interactive Threshold Algorithm Standard 24-2) using a Humphrey Field Analyser (HFA) [Carl Zeiss Meditec, Dublin, CA]. The best sensitivity values at each location of the monocular VFs were merged to construct an integrated visual field (IVF) [12, 13].

Participants completed the EuroQol-5 dimension (EQ-5D) questionnaire and the Ten-Item Personality Inventory (TIPI) at the start of the study in a face-to-face interview. EQ-5D [14] is a five-item measure designed to measure general health. The items are scored either 1 (no problems), 2 (some problems), or 3 (severe problems) on the domains of mobility, self-care, usual activities, pain/discomfort, and anxiety/depression. The individual 1-digit item scores are combined into a 5-digit number which describes health state. For example, a score of 12112 indicates a participant has some problems with self-care and anxiety but no other perceived problems. The TIPI [15] estimates levels of extraversion, agreeableness, conscientiousness, emotional stability, and openness to experience. The scale consists of 10 items, each scored on a 7-point Likert scale from 1 (disagree strongly) to 7 (agree strongly).

### 2.2. Web-Based Monitoring and Diary Tool

**Figure 1:**
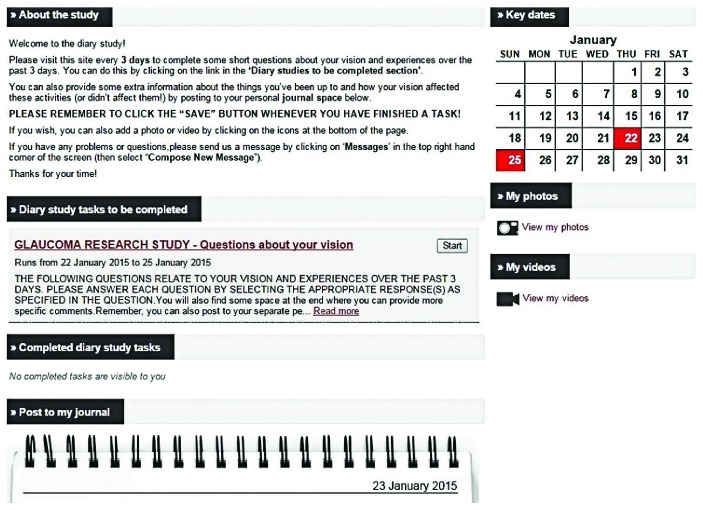
A screenshot of the web-based diary tool used by the participants. The page is split into “to be completed” and “completed” tasks.

The participants were introduced to the web platform at a face-to-face baseline visit and were provided with a unique login. The web platform was designed to be user friendly and easy to navigate (Figure 1). The participants were provided with a guidebook, which gave instructions about using the web tool.

The participants were asked to complete a set of bespoke “symptom monitoring” questions every three days. We asked how much driving, walking, searching for objects, using a computer, watching television, and eating and drinking were affected by glaucoma. These questions were scored on a 5-point Likert scale from “not at all” to “very much.” A summary score at each time point was generated (5 (no symptoms) and 45 (maximum symptoms)). The participants were sent automatic email prompts every three days as a reminder to complete the questions.

The participants were also invited to complete a written diary documenting any aspect of their glaucoma that they felt would be helpful to record. They could do this by typing directly into the web-based tool as frequently as they wanted to and could even upload photographs. This would be recorded by time and date. Again, they were prompted by an automatic email every three days.

### 2.3. Study Evaluation

**Figure 2:**
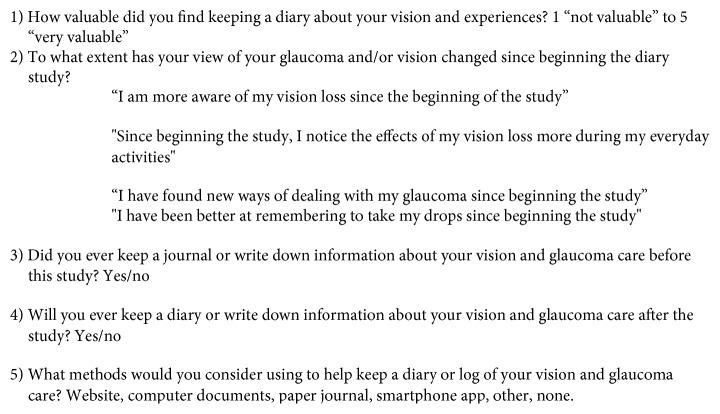
Study evaluation questions given to the participants at the end of the study.

The participants were asked to complete a series of questions (see Figure 2) about the usefulness of the exercise at the end of the eight-week study period.

### 2.4. Analysis

The composite symptom scores for each time point were used to plot change in symptom awareness over the course of the study. Individual personality traits for each participant were compared to the mean scores on the TIPI in a cross-sectional sample of the UK population (Table 1) [16]. The frequency of words written was used as a proxy for the level of diary usage. Univariate association between diary use and scores on personality traits was explored using Spearman's rho. The results from the evaluation questionnaire were assessed with simple summary statistics.

The information from the online diary tool was analysed using thematic analysis [17]. The lead researcher (LM) collated raw diary responses from each participant. The research team manually worked through each data set and highlighted sections of text that applied to the patients' glaucoma symptoms. These sections of text were grouped into themes.

## 3. Results

The participants (50% male) had a median age of 70 years (interquartile range (IQR) 66 to 76). The participants were from different regions of the UK and were educated to a minimum of high school level. All participants were married or living with a long-term partner.

A summary of patients' vision and baseline data is given in Table 2. Humphrey mean deviation (MD) in the better eye (BEMD) was used as a proxy measure for glaucoma disease severity. BEMD ranged from early to advanced, with median (IQR) BEMD −9.1 dB (−6.1, −13.4). Five participants had BEMD worse than −12 dB, and this level is sometimes described as advanced VF loss [18].

### 3.1. Symptom Monitoring

**Figure 3:**
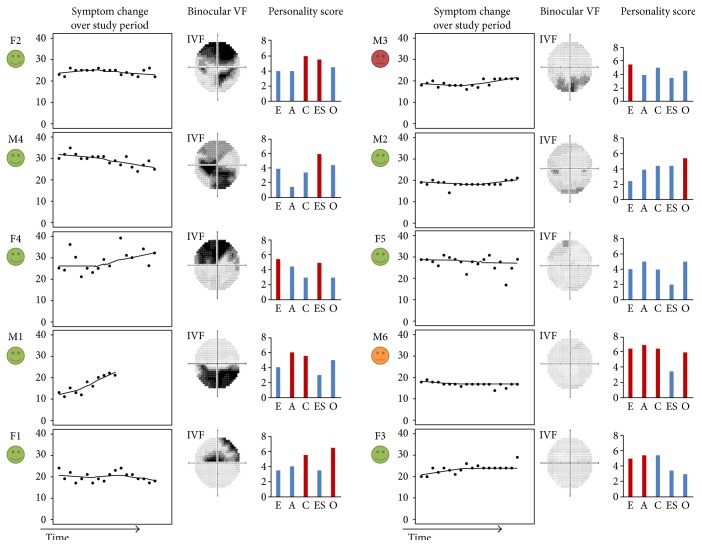
Shows results from 10 participants ordered according to the severity of binocular visual field loss. *From left to right*: face indicates self-response to review question about the value of the self-monitoring exercise; time series plot shows a composite visual symptom score recorded over a study period of 8 weeks. Binocular visual field is shown as grey scale of integrated visual field [5]. Individual bar chart indicates response to Big Five Inventory (BFI) personality questionnaire (E: extraversion, A: agreeableness, C: conscientiousness, ES: emotional stability, and O: openness). Red bars indicate that trait is significantly different from a reference population. For example, volunteer M6 had four significant personality traits.

The completion rate of the symptom-monitoring questions (96% over the eight-week period) was remarkably good. Composite symptom scores (from 5 to 45) for each time point were used to plot individual change in symptom awareness over the study period. Loess curves were fitted to the data points in order to illustrate any “trend” in symptom awareness during the study period [19] (see Figure 3()). These trends are purely illustrative given the short follow-up period.

The participants were remarkably well engaged with the diary entry tool. The median (IQR) number of diary words recorded per patient was 1858 (703, 4094) over the 8-week period.

Six participants reported higher levels of extraversion and openness to experience than the UK sample. Emotional stability was weakly correlated (rho = 0.39; *p* = 0.05) with the uptake of the diary exercise (number of words written in the diary exercise). There were no other statistically significant associations, but the sample size was very small.

### 3.2. Qualitative Analysis

Four main themes emerged from the thematic analysis at a semantic (explicit) level.

#### 3.2.1. Frustration

The participants often reported a feeling of frustration regarding their impaired ability to complete tasks because of their vision.

*It is very difficult to describe what it's like except that I know that my vision is not the same as it was a few years ago, it's not good and it's not right*. (F2)

Some participants felt frustration at themselves, describing that they should be able to complete certain tasks such as reading.

*As reading has become less pleasant, the piles of items waiting to be read tend to build up. Must try harder!* (F3)

*Not driving - wouldn't feel safe. Extremely difficult to read & shop. Getting very bad tempered & frustrated after almost 2 weeks of this.* (F5)

#### 3.2.2. Anxiety and Cessation of Activities

Some participants reported that they had stopped performing certain activities due to fears associated with their vision loss. Some of the instances of avoidance behaviour were preplanned.

*I find it difficult to see in the dark these days as I struggle where there is very little contrast. I have stopped driving at night but live in an urban area that is reasonably well served by public transport.* (F2)

There were also instances that appeared to be triggered by situational anxiety.

*During the night I started worrying about coping with trains and planes on my own and where I'd be able to find somewhere to rest up during Monday, as the only flight was very early. I felt so awful by Sunday morning that I decided I'd have to stay at home. So much for thinking I am back to normal...*. (F3)

#### 3.2.3. Social Support

The participants in this sample discussed social support networks mostly in a positive light but sometimes reported feeling guilty at having to rely on a partner for social support and feared becoming a burden.

*[Name omitted] drove me there but didn't come on the walk herself – I always feel a bit guilty about this...*. (M6)

*I don't like to rely on my partner for lifts but he often obliges. I will go out on foot with my trusty torch where necessary*. (F2)

The participants reported strong social support networks, including partners and friends, and emphasised the importance of professional support groups.

*IGA AGM was very much worthwhile attending. Loop system was working well so I could hear clearly. Particularly interested in all the research going on, DVLA [Driver and Vehicle Licensing Agency] aspect most relevant*. (F4)

Social support networks seemed to consist of different people for different participants; one reported a lot of activity involving friends, but some only talked about their partners. Regardless of who the network consisted of, participants spoke about the importance of their social support network understanding their glaucoma-related issues.

*I wouldn't have recognized him if he hadn't spoken – that sort of non-acknowledgement can probably seem rude to anyone who doesn't know about your glaucoma (I did apologize to him using the glaucoma excuse).* (M2)

Some also identified social activities as an important “distraction” factor.

*I'm not one for staying in bed but would prefer to keep active. Not up to my usual standard but still enjoyed the session. Didn't have time to ponder on how I felt and how my eyes were affected.* (M6)

#### 3.2.4. Clinician Trust

The participants described different aspects of their glaucoma care in their diary entries. Most participants indicated that they had high levels of trust and a helpful dialogue with at least some of their care team.

*Just glad my glaucoma was picked up when it was. If this is the sight I have 'for ever' whatever that means for me - then I am very grateful to have been looked after in the way I have been.* (F1)

There were very few participants who reported negative aspects of care, although some participants reported concern regarding interactions with professionals during their glaucoma care which led to mistrust.

*Opticians, new varifocals on order, titanium, bit pricey @ 640. But prefer to stick with local independent opticians. As one of larger chains, in my view, “missed” evidence of Glaucoma in its early stages when I complained that right eye vision through their new specs/lens provide was slightly inferior to left. This goes back some 8 years.* (M2)

Overall, the participants in this study reported having very positive relationships with their clinicians.

### 3.3. Evaluation of Study

Overall, the participants reported that they found the diary exercise valuable, with eight out of ten participants rating the exercise “valuable” or “very valuable.” One participant did not engage with the diary exercise and rated it not valuable at all. One participant rated the exercise neutral.

Interestingly, eight participants said they felt more aware of their vision loss and its effects since the beginning of the study. Only two of the ten participants felt that the intervention improved their medication adherence. Three participants felt that they had developed new ways of dealing with their vision loss.

Four participants said that they were more likely to keep an independent diary about their vision after completing the eight-week diary exercise. From the options given in the evaluation questions (see Figure 3()), five participants said they were most likely to use a web-based or computer-based diary tool.

The participants' experiences of the diary exercise were mostly positive. The participants generally felt that they received benefit from the diary exercise and that they would continue to benefit from using the process in the future.

*Thank you for asking me to take part in this research. No-one else knows the hassles I have mentioned, many others have bigger daily problems to cope with, so mine are trivial in comparison*. (F4)

Although the majority of comments were positive, one participant reported negative feelings.

*I don't think my sight is any worse than it was a few weeks ago, only that I am more focused on it. I am not sure that this is a good thing because it makes me more aware of problems when I would normally just deal with them or ignore them*. (F2)

## 4. Discussion

A group of self-selected volunteer patients, with a range of disease severity and personality types, adhered remarkably well to using a web-based diary tool to monitor their glaucoma symptoms. The participants were able to report their own symptoms with remarkable regularity, yielding plots of how their symptoms were potentially changing over time. Most participants felt more aware of their vision loss after taking part in the exercise. Themes emerging from the qualitative synthesis of the diary entries were related to behavioural aspects that might be overlooked in typical patient-clinician consultations. We speculate that aspects of a patient's quality of life affected by glaucoma (frustration and anxiety) could be flagged by an online monitoring tool and then assessed in clinical consultations.

An investigation of the feasibility of self-monitoring symptoms of glaucoma has not been done before. Our study therefore represents new knowledge as it has at least demonstrated how this might be feasible in a group of volunteer patients. Research into surveillance of glaucoma away from the clinic has, for example, focused on monitoring intraocular pressure and aids for improving adherence to treatment [20–23]. Here, we have shown that this approach might be useful in recording between clinic visit PROMs. Self-monitoring techniques have been shown to play a useful role in patient care in other chronic conditions [11, 24, 25]. The volunteers in our study were remarkably positive about the idea of self-monitoring. This may be related to the volunteer's personalities. For example, six participants reported higher levels of extraversion and openness to experience than a reference standard.

A number of patients in our study reported feeling anxious about their glaucoma. A higher prevalence of anxiety disorders has been demonstrated in other chronic conditions [26, 27]. Patients also reported frustration at losing their normal functional abilities. Evidence from other eye diseases has found links between loss of functional abilities and frustration [28]. Negative feelings likely have an impact on a patient's self-efficacy, and if they are not identified and addressed, patients may be more likely to develop depression [29]. An online monitoring tool may allow some patients to articulate these anxieties, and this could be clinically useful in the management of glaucoma.

The results from this study hint at important clinical applications, and we speculate on these briefly now. Evidence suggests that PROMs such as the ones used in this study, as well as self-monitoring exercises, provide important clinical information about patients which act as part of a collaborative management plan in chronic illness [30]. Many patients may not get an opportunity to discuss their condition during clinic appointments [31, 32]. A diary tool allows patients to use reflective thinking in order to pinpoint difficulties with their condition. For example, one participant in the study reported that she felt her problems were “trivial” compared to others and chose not to share them. Plotting self-reported symptoms, using an appropriate tool, could have the same motivational behavioural effect as measuring daily steps as a measure of exercise [33]. This might be useful in terms of engagement and adherence with treatment.

Participants in this study provided a substantial amount of written information about their psychological well-being which may not previously have been shared with clinicians. Patients may be less likely to disclose psychological distress with clinicians due to fear of stigmatisation or involvement of mental health services [34, 35]. Interestingly, some evidence suggests patients are more likely to disclose information of a sensitive nature if they are able to do so using technologically advanced methodology, such as through a web-based tool [36, 37]. An online diary may therefore yield more information about a patient's psychological well-being when compared to a hospital consultation, and this should be investigated further.

One patient concluded that a constant focus on monitoring symptoms led to negative feelings and experiences. This is very noteworthy. Previous research has suggested that private self-focus and rumination are associated with depression and generalised anxiety in some people [38]. This observation would be important to consider in the development of the idea of self-monitoring symptoms. Moreover, the diary tool may have been making patients more aware of problems with their vision and this has significant implications that need to be considered in a future study. It would, for example, be interesting to integrate an exercise such as the one we have carried out with measures of adherence to treatment, which is a serious issue in glaucoma management. Interestingly, only two of the ten participants in our study felt that the intervention improved their medication adherence.

The experimental design of our study had several strengths, such as the combination of use of personality testing and symptom-monitoring questions. Of the ten participants, only one chose not to use the qualitative diary tool throughout the course of the study; however, this participant did complete the symptom-monitoring questions. The study used a multifaceted approach which allowed participants to engage only with the parts of the exercise that they were comfortable with. The web pages were well designed, and all data was safely and securely captured.

There are also several limitations to our study. The study sample was small, and the glaucoma profile of the patients was very varied; this prevents us from drawing real conclusion other than proving the practical feasibility of the approach. Volunteers were self-selected and motivated. Volunteers had good levels of education and were sufficiently engaged with their glaucoma because, for example, they belong to a patient organisation. We do not know if adherence to the exercise would be so good in another population.

In conclusion, volunteer patients, with a range of disease severity and personality types, adhered remarkably well to using a web-based diary tool to monitor their self-reported glaucoma symptoms. A web-based diary intervention for the self-monitoring of glaucoma may therefore be practical. Future work should examine the feasibility of this approach in larger groups of patients with broader methods of recruitment and examine if it can change behaviour or be clinically useful. The monitoring tool must be carefully designed in order to ensure that the participants are benefitting, and it is not increasing anxiety.

## Figures and Tables

**Table 1:** 

	Extr.	Agre.	Consc.	Emo stab.	Open.
Population mean	9.11	10.12	10.44	9.05	10.11
M1	8	*12*	*11*	6	10
M2	5	8	9	9	*11*
M3	*11*	8	10	7	8
M4	8	3	7	*12*	9
M6	*13*	*14*	*13*	7	*12*
F1	7	8	*11*	7	*13*
F2	8	8	*12*	*11*	9
F3	*10*	*11*	*11*	7	6
F4	*11*	9	6	*10*	6
F5	8	10	8	4	10

The table demonstrates the raw Ten-Item Personality Inventory data for the study sample. Scores from left to right: extraversion, agreeableness, conscientiousness, emotional stability, and openness. Items in italic denote that the score is above reference population mean [16].

**Table 2:** A summary of patients' vision and baseline data.

	Years since diagnosis	Binocular visual acuity (LogMAR)	Binocular contrast sensitivity	Best eye HFA mean deviation (dB)	EQ-5D general health
M1	21	−0.2	1.95	−13.7	11111
M2	5	0	1.5	−7.9	11111
M3	26	−0.02	1.65	−5.5	11211
M4	23	−0.1	0.9	−17.4	21111
M6	25	0	1.95	−11.4	11111
F1	29	−0.1	1.2	−9.2	11111
F2	11	−0.1	1.35	−19.4	11211
F3	6	0	1.95	−2.2	11121
F4	15	0.1	1.35	−13.6	21211
F5	15	0	1.35	−9.0	11221
